# Calibration of the Global Flood Awareness System (GloFAS) using daily streamflow data

**DOI:** 10.1016/j.jhydrol.2018.09.052

**Published:** 2018-11

**Authors:** Feyera A. Hirpa, Peter Salamon, Hylke E. Beck, Valerio Lorini, Lorenzo Alfieri, Ervin Zsoter, Simon J. Dadson

**Affiliations:** a Oxford University, School of Geography and the Environment, Oxford, UK; b European Commission Joint Research Centre, Disaster Risk Management Unit, Ispra, Italy; c Princeton University, Civil and Environmental Engineering, Princeton, USA; d European Centre for Medium-Range Weather Forecasting, Reading, UK

**Keywords:** Flood forecasting, GloFAS, Global hydrology, Early warning system, Model calibration

## Abstract

This paper presents the calibration and evaluation of the Global Flood Awareness System (GloFAS), an operational system that produces ensemble streamflow forecasts and threshold exceedance probabilities for large rivers worldwide. The system generates daily streamflow forecasts using a coupled H-TESSEL land surface scheme and the LISFLOOD model forced by ECMWF IFS meteorological forecasts. The hydrology model currently uses a *priori* parameter estimates with uniform values globally, which may limit the streamflow forecast skill. Here, the LISFLOOD routing and groundwater model parameters are calibrated with ECMWF reforecasts from 1995 to 2015 as forcing using daily streamflow data from 1287 stations worldwide. The calibration of LISFLOOD parameters is performed using an evolutionary optimization algorithm with the Kling-Gupta Efficiency (KGE) as objective function. The skill improvements are quantified by computing the skill scores as the change in KGE relative to the baseline simulation using *a priori* parameters. The results show that simulation skill has improved after calibration (KGE skill score > 0.08) for the large majority of stations during the calibration (67% globally and 77% outside of North America) and validation (60% globally and 69% outside of North America) periods compared to the baseline simulation. However, the skill gain was impacted by the bias in the baseline simulation (the lowest skill score was obtained in basins with negative bias) due to the limitation of the model in correcting the negative bias in streamflow. Hence, further skill improvements could be achieved by reducing the bias in the streamflow by improving the precipitation forecasts and the land surface model. The results of this work will have implications on improving the operational GloFAS flood forecasting (www.globalfloods.eu).

## Highlights

• GloFAS was calibrated using daily streamflow data from 1287 stations worldwide.
• Evolutionary Algorithm with parallel co-evolution of multiple simulations was used.
• Parameters will be used in operational flood forecasting (www.globalfloods.eu).
• Bias reduction in the forcings is needed to further improve the forecast skill.

## 1 Introduction

Flood early warning systems (FEWS) are principal components of disaster risk management since they provide a unique opportunity to identify upcoming flood hazards ahead of their occurrence. However, the effectiveness of early warning systems depends on the skill with which the occurrence and severity of floods can be predicted. In recent years, global-scale operational flood forecasting systems have emerged as promising support tools for disaster preparedness and response worldwide, and major effort has been dedicated to improving the skill of such systems (see reviews in Emerton et al., 2016, Hirpa et al., 2018).

Flood forecast skill is primarily affected by: (i) model errors due to incomplete representation of physical processes and inaccurate parameterization, (ii) uncertainty in the model initial conditions, and (iii) errors in the meteorological forcing (e.g., Fekete et al., 2004, Biemans et al., 2009, Pappenberger et al., 2011, Nasonova et al., 2011, Müller Schmied et al., 2014, Sperna Weiland et al., 2015). Several studies have investigated the relative contributions of the meteorological forcing (precipitation), initial conditions, model structure or parameters uncertainty to the errors in the streamflow simulations (e.g., Fekete et al., 2004, Li et al., 2009, Materia et al., 2010, Nasonova et al., 2011, Elsner et al., 2014, Sampson et al., 2014, Sperna Weiland et al., 2015, Döll et al., 2016). Even though precipitation uncertainty was generally identified as the largest contributor to the error in the simulated streamflow (e.g., Sperna Weiland et al., 2015, Beck et al., 2016), calibration of global-scale model parameters has been demonstrated to improve streamflow simulations (e.g., Werth and Güntner, 2010, Müller Schmied et al., 2014, Beck et al., 2016).

Calibrating a global-scale hydrological model is a challenging task mainly due to the high computational demand and the limited availability of reliable streamflow observations (e.g., Bierkens, 2015). Hence, previous works have been limited to small number of basins, performed at a relatively larger catchments and/or temporal scales (e.g., monthly), or using a proxy streamflow data derived from satellite observations (e.g., Werth and Güntner, 2010, Sperna Weiland et al., 2015, Revilla-Romero et al., 2015a, Revilla-Romero et al., 2015b). The increasing advances in the computational power and progressively improving calibration algorithms present an opportunity to perform model calibration at the global scale and higher spatial and temporal resolutions.

Large-scale hydrological modelling using grid-based hydrological routing model coupled with a land surface model has been used for generating streamflow for large river basins or at continental and global scales (e.g., Goteti et al., 2008, Decharme et al., 2010, Dadson et al., 2011, Alfieri et al., 2013, Li et al., 2013, Wu et al., 2014). In the coupled modeling systems the land surface model generates runoff from meteorological forcing by calculating the water balance, and a routing model is used to calculate the flow in river channels.

The Copernicus Global Flood Awareness System (GloFAS, Alfieri et al., 2013; www.globalfloods.eu) produces ensemble streamflow forecasts and threshold exceedance probabilities for large rivers around the world. The system employs a coupled land surface scheme and flow routing model to generate operational forecasts at daily time steps and 0.1° grid resolution. The land surface scheme, referred to as Hydrology Tiled ECMWF Scheme for Surface Exchanges over Land (H-TESSEL; Balsamo et al., 2009) and used operationally in the Integrated Forecast System (IFS), computes the surface and subsurface runoff. A simplified version of LISFLOOD (Burek et al. 2013) is used for routing the runoff produced by the land surface scheme through the river network and computing the groundwater fluxes. GloFAS serves as a primary mechanism of support for flood early warning in regions without local FEWS and provides complementary flood forecast information for areas with alternative systems such as national or continental FEWS. Much effort has been directed at improving GloFAS forecast skill (e.g., Zajac et al., 2017, Hirpa et al., 2016, Revilla-Romero et al., 2015a, Revilla-Romero et al., 2015b, Zsoter et al., 2016), however calibration of the hydrological model has not been previously performed.

In the present study we calibrate the hydrological model parameters underpinning the operational GloFAS using daily streamflow observations from 1287 stations across the globe. The significant contribution of this calibration work lies in its application to global-scale operational flood forecasting system hence can help improve the flood early warning through enhanced flood forecast skill. Furthermore, the calibration covers hundreds of river basins with a wide range of climate including snow-dominated boreal basins, equatorial basins with high runoff, and dry basins with low runoff. To accommodate the high computational demand of global-scale calibration of a distributed hydrological model, an Evolutionary Algorithm with co-evolution of multiple simulations running in parallel was used (e.g., Deb et al., 2002, Tang et al., 2006).

The main objectives of the work are to improve the operational flood forecasting skill by calibrating the flow routing and groundwater model parameters, and to quantify the contribution of the flow routing model parameters uncertainty to the streamflow forecast skill. We quantify the skill gained as the result of model calibration by comparing to baseline simulations produced using default parameter sets determined based on the literature (see Feyen et al., 2007). The transferability of parameters to another period was evaluated using a different time period from calibration. While calibration of the routing model alone is unlikely to remove all the errors in the streamflow simulation, we aim to reduce the relative contribution of the parameter error to the streamflow forecast uncertainty.

The remainder of this paper is organized as follows. The runoff routing model and the calibrated parameters are described in Section 2. The calibration method and data are presented in section 3, followed by the evaluation method in Section 4. Results, discussions and conclusions are presented in 5, 6, 7 respectively.

## 2 LISFLOOD model and parameters

LISFLOOD is a distributed hydrological model composed of sub-models capable of separately simulating different hydrological processes. In GloFAS, the model is run at 0.1° resolution globally and at daily time step. The sub-models are used for the simulation of groundwater storage, groundwater flow, and flow routing into and through river channels. The groundwater storages and transport are represented using two interconnected groundwater zones each consisting of a linear reservoir (Burek et al., 2013). The outflow from each zone is estimated based on the water storage and time constant of the reservoir. The two reservoir time constants, the percolation from upper to lower zone, and a loss from lower zone to deep groundwater are calibrated parameters (see Table 1).

**Table 1:** A list of calibrated LISFLOOD parameters. The GloFAS operational model currently uses the default parameter values.

Parameter name	Description [unit]	Parameter values		
		Lower bound	Upper bound	Default value
*UpperZoneTimeConstant*	Time constant for water in upper zone [days]	3	40	10
*LowerZoneTimeConstant*	Time constant for water in lower zone [days]	40	500	200
*GwPercValue*	Maximum rate of percolation going from the upper to the lower groundwater zone [mm/day]	0.01	2	0.5
*GwLoss*	Maximum loss rate out of lower groundwater zone expressed as a fraction of lower zone outflow [−]	0	0.5	0
*CalChanMan*	A multiplier applied to Channel Manning's coefficient *n* [−]	0.1	15	4
*LakeMultiplier*	A multiplier to adjust the lake outflow width parameter [−]	0.5	2	1
*adjust_Normal_Flood*	Adjusts the balance between normal and flood storage of a reservoir [−]	0.01	0.99	0.8
*ReservoirRnormqMult*	A multiplier to adjust the magnitude of the normal outflow from a reservoir [−]	0.25	5	1

There are three routing components in LISFLOOD: i) routing the surface runoff into stream channel, ii) subsurface runoff to the channel, and iii) flow routing through the stream channel. The surface runoff and in-channel routing are performed by solving kinematic wave equations (Chow, 1998). For each model pixel at every time step (here set to four hours), a 4-point implicit finite-difference solution of the kinematic wave equations is applied to compute the flow of water to the nearest downstream channel (Burek et al., 2013). The subsurface runoff routing into the stream channel is modeled differently. The total outflow from upper and lower groundwater zones at a given time step is routed to the nearest downstream channel pixel as a scaled sum of the outflow from upper and lower groundwater zones.

The routing operations, implemented using the PCRaster software (http://pcraster.geo.uu.nl/), require information about stream channel characteristics such as channel length and gradient, flow width and depth, and Manning’s roughness coefficient. For GloFAS, the river network map, the flow direction map, the upstream area, and the flow length were obtained from the global river network database (Wu et al. 2012). The channel gradient between adjacent pixels was estimated as a ratio of the elevation change to the flow length. The river widths were taken from the Global Width Database for Large Rivers (GWD-LR; Yamazaki et al. 2014). The bankfull water depth was empirically estimated using long-term average discharge by applying Manning’s equation.

In GloFAS, a total of 463 large lakes (>100 km^2^) and 667 reservoirs are incorporated to the LISFLOOD model (see Zajac et al, 2017 for the locations). The attributes of the lakes and reservoirs were obtained from global databases (Lehner and Döll, 2004, , 2009, Lehner et al., 2011). Outflow from a lake is estimated using its relationship with the lake level using weir equation (Bollrich, 1992). The lake level variation is estimated from the change in lake storage over time (Zajac et al., 2017). Each reservoir is simulated based on its relative filling levels and design parameters such as total storage capacity, storage limits and outflow requirements. The reservoir parameters were estimated from the metadata obtained from global database. However to improve the simulation skill we calibrate to adjust the balance between normal and flood storage limits of a reservoir, and the normal outflow from a reservoir (Table 1).

Irrigation water use is modeled as a monthly withdrawal from discharge in the river network, but any potential outtake from groundwater, precipitation or soil water is not accounted for. Open water (e.g., river channels and lakes) evaporation is estimated using the Penman–Monteith equation with the required variables extracted from ECMWF reforecast datasets consistent with the forcing data (see Section 3.2).

## 3 Calibration method and data

### 3.1 Calibration method

#### 3.1.1 Algorithm

We used an Evolutionary Algorithm (EA) for the calibration of the LISFLOOD model parameters. EA is a population-based optimization algorithm in which each individual (e.g, a vector of model parameters) in a large population represents a candidate solution for the optimization problem. The goodness of fit for each individual is evaluated based on selected objective functions, which are designed as either maximization (e.g, Kling-Gupta efficiency) or minimization (e.g., root mean square error) equations constrained by physically meaningful model parameter ranges. The basic principle of the EA is to modify and improve the population through evolution over a range of generations, and ultimately identify the best performing individual. The evolution mechanisms from one generation (parent) to the next (offspring) are inspired by biological evolution such as mutation, crossover and reproduction (Maier and Co-authors, , 2014, Nicklow et al., 2010). EAs have been previously used for calibration of hydrological and/or water resources models (e.g., Muleta and Nicklow, 2005, Kollat and Reed, 2005, Tang et al., 2006, Khu et al., 2008, Hadka and Reed, 2013, Maier and Co-authors, , 2014, Beck et al., 2016).

In EA, the evolutionary loop starts by generating offspring (λ) from the population (µ). In this work this was performed by varying the population using a combination of a blend crossover (90%) and Gaussian-based mutation (10%) algorithms (Fortin at al., 2012). Determining the fraction of the population varied by crossover or mutation is part of the algorithm design (De Rainville et al., 2014), and its impact on the accuracy of the optimum solution can be investigated in a separate work. The offspring generation is followed by offspring evaluation in order to determine the best performing individuals. This was performed though model simulation using the newly generated model parameters. We applied a Non-dominated Sorting Genetic Algorithm (NSGA-II; Deb et al., 2002) for the selection of the best performing parameters from a mixed set of parents and offspring (µ+λ). The evolutionary loop continues until a stopping criterion is met. EAs have been shown to be one of the most efficient and effective algorithms in hydrologic calibration and particularly the NSGA-II algorithm was previously found to be superior to other algorithms with its rapid convergence (Tang at al., 2006).

#### 3.1.2 Implementation

The Kling-Gupta efficiency (KGE; Gupta et al., 2009) was selected as the objective function for the calibration. Due to the high computational demands of the gridded LISFLOOD model at global-scale, it was necessary to limit the total number of model simulations during the calibration exercise. Here a combination of improvement based criteria (i.e., improvements in the objective functions) and exhaustion-based criteria (fixed number of generations) was employed for stopping the calibration algorithm. Using a selected number of basins, we first determined the rate of change of the objective function (KGE) with the number of generations in the evolutionary algorithm. Typically there is a fast improvement rate in the initial steps of the calibration, and then a slower rate for longer generations until there is no further skill gain for any additional generation after a convergence to an optimum parameter set. Since there was minor improvement beyond 10 generations for most of the test basins we determined that the maximum number of generations for the calibration runs to be 15. For the improvement based criterion, we determined that if, after 5 generations the KGE improvement for four consecutive generations is less than 0.001, the algorithm stops. In other applications, if the computational time is not a concern, one can run for a larger number of generations before obtaining the optimal parameter sets. Moreover, it might be desirable to implement several other stopping criteria (see, Marti et al., 2009).

The EA algorithm was implemented using Python programming language and is publicly available as Distributed Evolutionary Algorithm in Python (DEAP; Fortin et al., 2012, De Rainville et al., 2014; https://github.com/deap/deap). The µ and λ values are set to 12 and 24 respectively, and, as mentioned previously, the probability that an offspring is produced by crossover (mutation) was set to 0.9 (0.1). Multiple parallel runs in a given generation are performed by distributing on multiple cores on a Window® PC. The calibration was run for a maximum of 372 simulations (15 generations, µ = 12 and λ = 24), unless the improvement criterion was met earlier, for each calibration station before stopping. For LISFLOOD, these simulations take, on average, approximately six hours for each calibration station on a PC with 12 cores. For stations located in the same river basin, the calibration was iteratively performed from upstream to downstream (in ascending catchment area) using the streamflow from the calibrated upstream subbasin(s) as inflow for the calibration of the next interstation area. This approach accounts for geomorphologic connections along the drainage network and was shown to improve model performance over independent single-site calibration (Wi et al., 2015, Xue et al., 2016).

### 3.2 Forcing data

For this work, the surface and subsurface runoff from ECMWF reforecast were used as input to the LISFLOOD model. The runoff fields were produced by the ECMWF land surface scheme, HTESSEL, which performs the energy and water balance calculations over land and water grids (e.g, Balsamo et al. 2009). The reforecast is created based on a retrospective run of the most recent version of the ECMWF’s Integrated Forecast System (IFS) which undergoes regular updates to improve the weather forecast (see, ECMWF, 2017). The purpose of the retrospective run is to generate long-term (20 years) datasets consistent with the operational weather forecasts (Alfieri et al., 2014). This makes the reforecast a suitable forcing data for the calibration of the operational LISFLOOD model parameters. The data record spans from June 1995 to June 2015, and due to frequent model updates it is based on multiple model cycles: Cycle 41r1 (July through March) and Cycle 41r2 (April through June), with horizontal resolutions of ∼32 km and ∼18 km respectively. The runoff fields are interpolated using nearest neighbour method from the native resolutions to 0.1° × 0.1° grids before they were used for forcing LISFLOOD.

### 3.3 Streamflow data

**Fig. 1:**
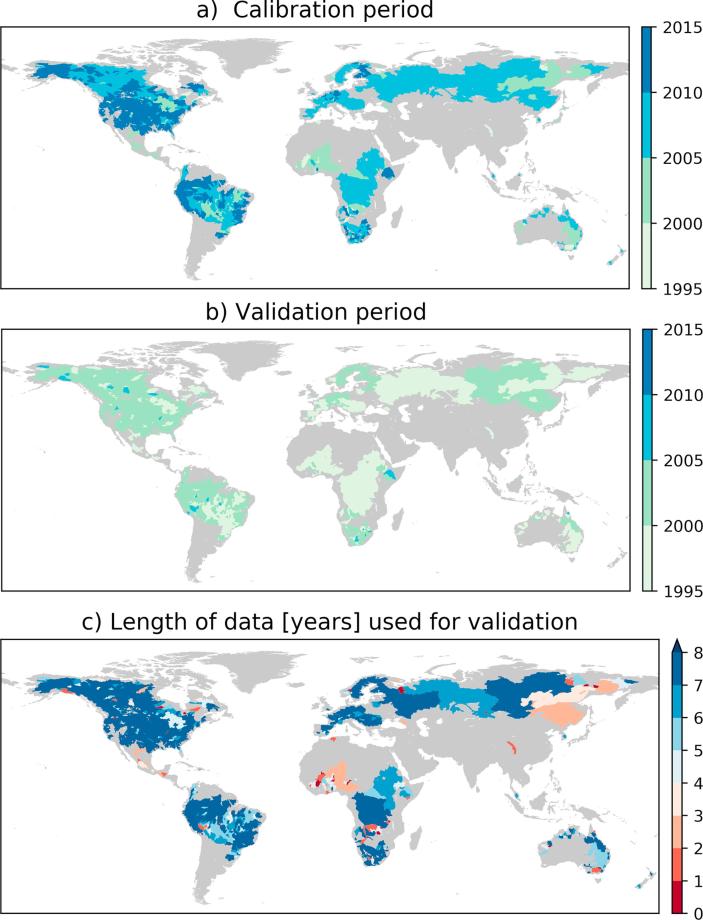
The median year of streamflow data used for model calibration (a), for validation (b) and the length of data available for validation (c). The most recent data available were used for calibration. Four years of data was used for calibration for those stations with validation period of less than 4 years, and the remaining stations have equal length of both calibration and validation periods.

Daily streamflow observations from 1287 stations around the world were used for the model calibration and evaluation. The catchment areas of the stations vary from 484 km^2^ to 4.8 × 10^6^ km^2^, with a median of 38,000 km^2^. The regional distribution of the stations is as follows: 41.6% are located in North America, 25.6% in South America, 14.1% in Europe, 10.9% in Africa, 4.5% in Australia and Oceania, and 3.3% in Asia. The Global Runoff Data Centre (GRDC) was the primary source of the streamflow data, but additional data from national hydrological services (e.g., South Africa, www.dwa.gov.za) and from the R-ArcticNet database for the Arctic region (http://www.r-arcticnet.sr.unh.edu/v4.0/AllData/index.html) were used. We only used stations with more than four years of data during the study period (1995–2015). For each station, the record was split into two for calibration and validation purposes (Fig. 1). If the record was shorter than eight years, four years were used for calibration and the remaining days were used for validation. Validation was not performed if the available data is less than 1 year. If the record was equal to or longer than eight years, half was used for calibration and half for validation. The most recent period was used for the calibration because the earlier forcing data have greater uncertainty.

## 4 Evaluation

**Fig. 2:**
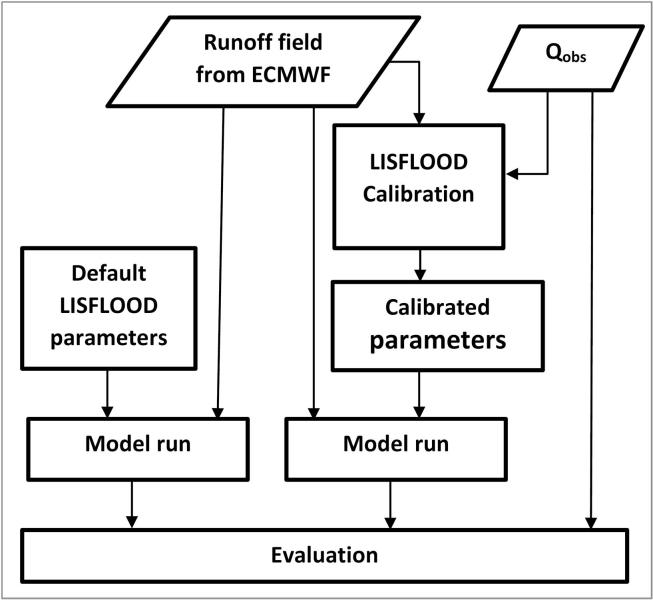
The schematics of the streamflow evaluation for the calibration and validation periods (see Fig. 1 for the time periods).

The evaluation was performed separately for the calibration and validation periods (see Fig. 2). For the calibration period, the improvement in streamflow simulations as a result of the calibrated parameters ($Q_{\mathrm{cal}}$) was assessed by comparing with a baseline simulation ($Q_{\mathrm{def}}$), which was generated using default model parameters. The Kling-Gupta efficiency (KGE), correlation (R), percent bias (B) and Nash–Sutcliffe efficiency (NSE) (Gupta et al., 2009) were used as evaluation metrics. The relative improvements were expressed as skill scores:

(1) $${\mathrm{KGE}}_{\mathrm{SS}}=\frac{{\mathrm{KGE}}_{\mathrm{cal}}-{\mathrm{KGE}}_{\mathrm{def}}}{{\mathrm{KGE}}_{\mathrm{perf}}-{\mathrm{KGE}}_{\mathrm{def}}}$$

where: ${\mathrm{KGE}}_{\mathrm{SS}}$ denotes KGE skill score; ${\mathrm{KGE}}_{\mathrm{def}}$ is KGE with default parameters; ${\mathrm{KGE}}_{\mathrm{cal}}$ is KGE with calibrated parameters; and ${\mathrm{KGE}}_{\mathrm{perf}}$ indicate KGE of a perfect simulation (=1). Positive (negative) ${\mathrm{KGE}}_{\mathrm{SS}}$values indicate improved (deteriorated) skill after calibration. The best ${\mathrm{KGE}}_{\mathrm{SS}}$ value is 1. For each case, the KGE was computed with reference to streamflow observations ($Q_{\mathrm{obs}}$). A similar skill score computation was repeated for R and NSE. In order to have a comparable range of skill scores (i.e., with the best score being 1), the B skill score (B_SS_) was computed using the absolute values of the percent bias as follows:

(2) $$B_{\mathrm{SS}}=\frac{{|B}_{\mathrm{cal}}\left|-{|B}_{\mathrm{def}}\right|}{{|B}_{\mathrm{perf}}\left|-{|B}_{\mathrm{def}}\right|}$$

where the *B*_perf_ (=0) is the ^B^ of a perfect simulation, *B*_cal_ is *B* after calibration and *B_def_* is *B* with default parameters. The second evaluation, during the validation period, was performed using streamflow observations from a different time period, to assess the temporal transferability of the calibrated model parameters.

## 5 Results

### 5.1 Streamflow with default parameters

**Fig. 3:**
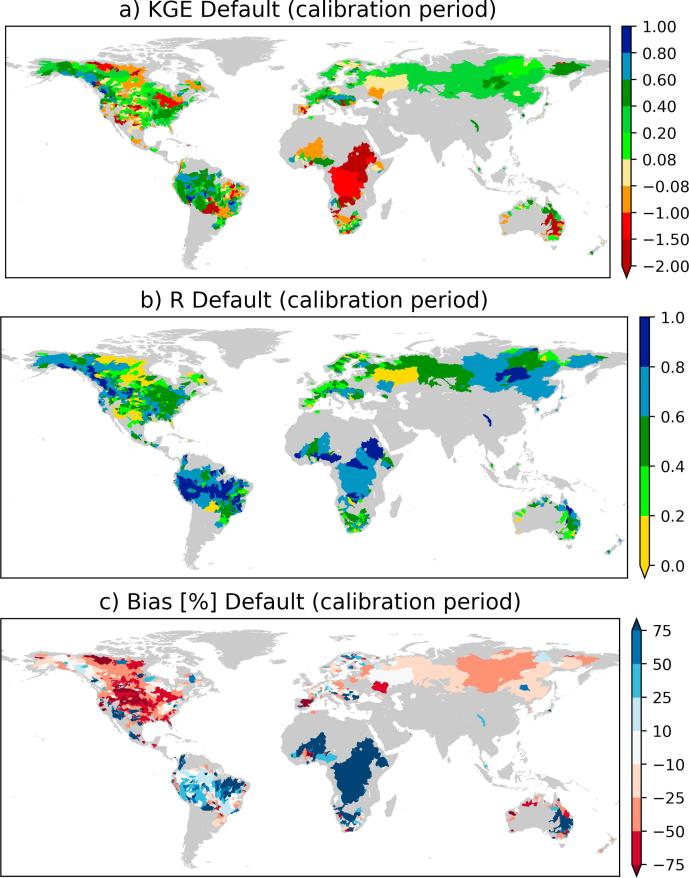
Performance of the baseline simulation (with default LISFLOOD parameters).

To evaluate the accuracy of the baseline streamflow simulation, we first compared it with observed streamflow data. The computed scores during the calibration period (KGE, B and R) show a large regional difference in the performance of the streamflow simulated with default parameters (Fig. 3). The KGE score is high (e.g., >0.2) in most parts of the Amazon, southeastern United States, Europe, northwestern North America and Russia. However, the KGE score is low (less than0) for most part of Africa, Midwestern United States and Australia. The regions with low KGE score also have large bias (positive or negative) in the simulated streamflow (Fig. 3b). Specifically, large underestimation in the United States and large streamflow overestimation in Africa (e.g., Nile, Congo, Niger basins) and Murray-Darling in Australia contributed to the low KGE score in those regions. Tropical basins have high daily streamflow temporal correlation (>0.6), but parts of North America have low temporal correlation (Fig. 3c). The streamflow overestimation in the tropics is consistent with previous reports that atmospheric models have a tendency of overestimating rainfall amounts (e.g., Beck et al., 2017, Trenberth et al., 2011, Kang and Ahn, 2015). The reason for the large streamflow underestimation in North America is, however, unclear since atmospheric models tend to have low bias in high latitudes.

### 5.2 Streamflow with calibrated parameters

#### 5.2.1 KGE skill gain

**Fig. 4:**
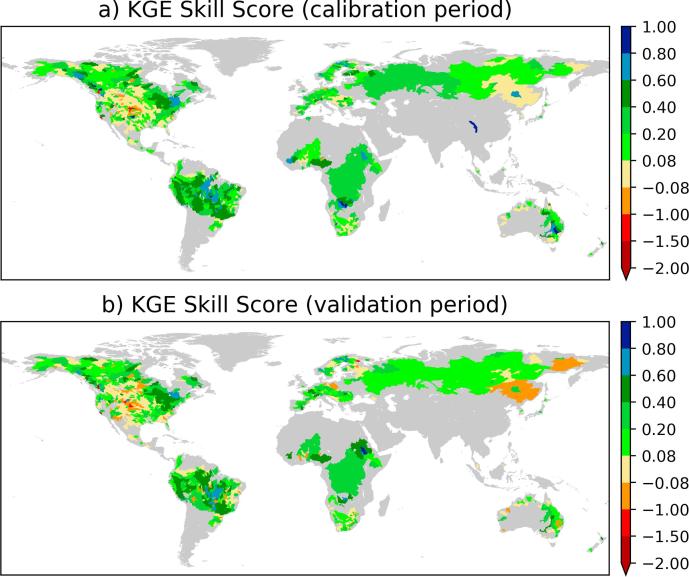
The KGE skill score during calibration (top) and validation (bottom) periods.

To quantify the improvements in simulated streamflow as the result of calibrating the LISFLOOD model parameters, we calculated the KGE skill score for each calibrated river basin both for the calibration and validation periods (see Fig. 4). The skill scores during the calibration period show that the streamflow skill improved due to calibration for the majority of the river basins. Overall, model calibration improved for 67% of the basins (with KGE skill score of >0.08) with a median KGE skill score of 0.15 (P90 = 0.46 and P10 = −0.002). However there was no skill gain (29% with KGE skill score close to 0) or skill loss (4% with negative KGE skill score, <−0.08) for the remaining basins after calibrating the LISFLOOD model parameters. If North America, where there was a large negative bias and a considerable number of skill loss after calibration was observed, is excluded the percentage of skill gain would increase to 77%, and the remaining stations will have 1% skill loss and 22% no skill.

The overall KGE skill score for the validation period is slightly worse than the one for the calibration period. Globally, calibrating the model parameters improved the performance for 60% of the stations; we found skill loss for 10% during the validation period. The median KGE skill score is 0.12 (P90 = 0.42 and P10 = −0.075). In a similar pattern to the calibration period, the skill score map shows that the majority of the skill loss occurred in North America.

#### 5.2.2 Regional breakdown

**Fig. 5:**
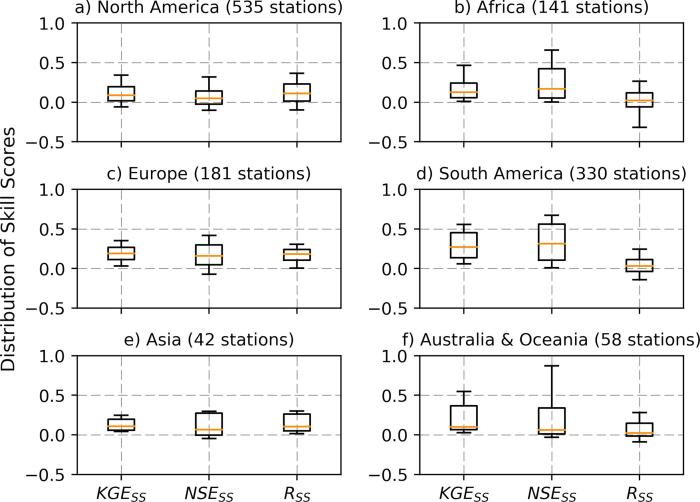
Regional variations of KGE, NSE and R skill scores (B skill score is presented below).

To investigate the regional variations and the impact of catchment area on the skill gain we present the regional breakdown of the KGE, NSE and R skill scores (Fig. 5). Results reveal that the skill score varies across regions. On the one hand, the KGE skill improved (KGE_SS_ > 0.08) for a significant number of sub-basins across all regions: 84% in in South America, 82% in Europe, 67% in Australia and Oceania, 62% in both Africa and Asia, and 52% in North America. This indicates that the calibration of the LISFLOOD model parameters improved the performance of the simulated streamflow; however the extent of the improvement varies across regions. On the other hand calibrating only the selected model parameters has limitations: there was no added skill (KGE skill score ∼ 0) compared to the baseline streamflow for 39% of basins in North America, 38% in Asia, 35% in Africa, 33% in Australia, 16% in South America, and 14% in Europe. Furthermore, there is a small number of basins with negative KGE skill score (e.g., 9% in North America). The median NSE skill score is similar to those of KGE across all regions, but it has more spread for most of the continents. The R skill scores show different patterns, notably in South America where the large KGE skill gain after calibration has not been reflected in R skill score. Possible influence of the inherent bias in baseline simulation on the skill scores is investigated in Section 5.3. While here we use the calibration period to examine the regional dependence of the skill score, a similar pattern was observed for the validation period.

#### 5.2.3 Separation by hydro-regions

**Fig. 6:**
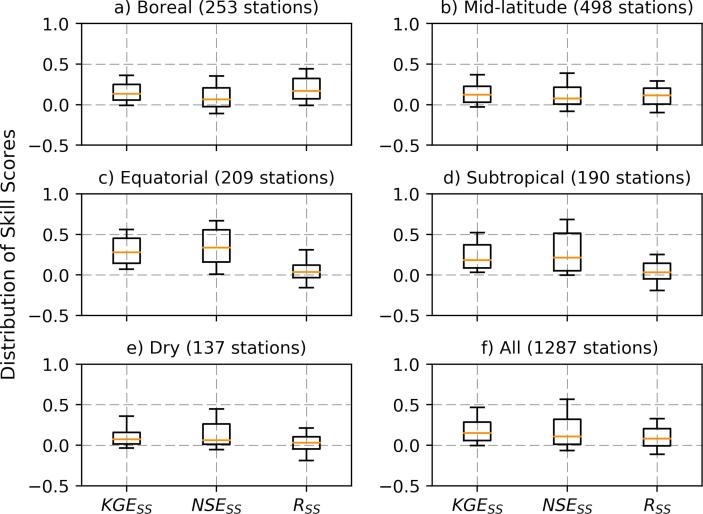
Distribution of skill scores classified based on hydro-regions (Meybeck et al., 2013).

To check if hydroclimate was a factor for the skill improvement, we show the calibration skill score variations with hydro-regions (see Fig. 6). The global river basins are classified by Meybeck et al. (2013) into hydro-regions (also called ‘hydrobelts’) based on mean annual runoff and temperature. According to the classification by Meybeck et al. there are five global hydro-regions: Boreal (mean annual temperature below 0 °C), mid-latitude (mean annual temperature between 0 and 15 °C and runoff between 150 and 750 mm), subtropical (mean annual temperature between 15 and 25 °C and runoff between 150 and 750 mm), equatorial (mean average temperature exceeding 20 °C and runoff > 750 mm), and dry (mean annual runoff less than 150 mm) regions. The results show that hydroclimate is an important factor with regards to the calibration skill improvement. The equatorial and subtropical basins showed the best skill score with almost all basins having a positive KGE skill score. While the large majority of basins in mid-latitude (primarily in North America as discussed above) have positive skill score, there are several basins with poor skill after calibration. Boreal and dry basins have large majority of basins with enhanced streamflow skill, but also a handful of basins with negative KGE skill score. The NSE and R skill scores also vary across regions. The equatorial and subtropical regions showed relatively higher NSE skill score and lower R skill score compared to other regions.

### 5.3 The impact of bias on the skill score

**Fig. 7:**
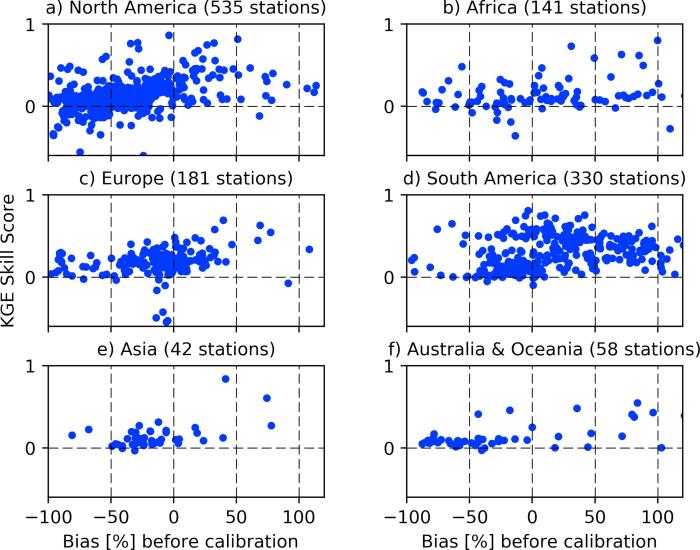
KGE skill score as a function of bias in the baseline simulations.

A potential bias in streamflow may not be adjusted with tuning the model parameters listed in Table 1. This is especially important when there is a negative bias in the runoff forcing in which the water deficit cannot be improved unless the runoff bias is first corrected. To examine weather runoff bias contributed to the calibration skill, we present the KGE skill score as a function of streamflow bias in the baseline simulation (generated with default parameters) for different regions (see Fig. 7). We found that the predominant stations with negative KGE skill score are those with negative bias in the baseline streamflow. Furthermore, almost all stations with positive bias in the baseline streamflow have improved KGE skill score. This is because positive streamflow bias can be corrected by tuning the groundwater percolation or loss parameter to transfer more water to the deep groundwater storage.

### 5.4 How do parameters adjust?

**Fig. 8:**
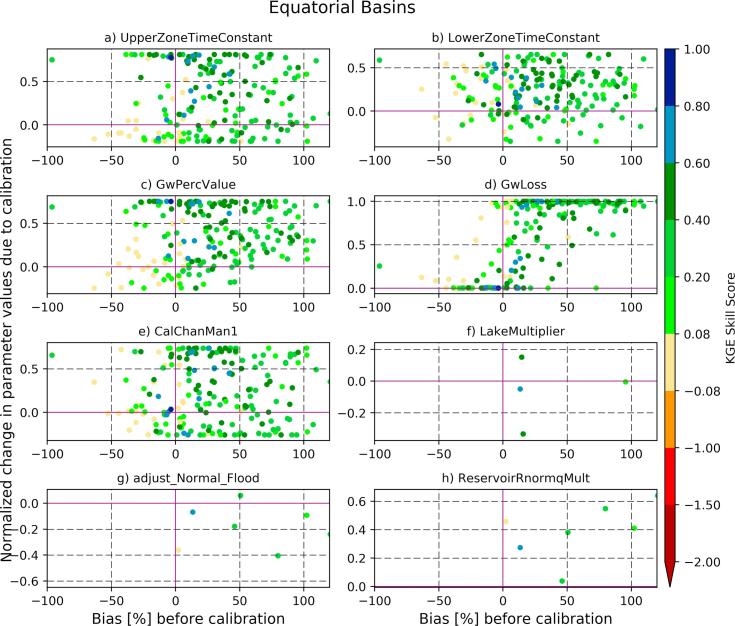
Impact of the change in model parameters after calibration on the KGE skill score. Each dot in the scatter plot represents a calibrated sub-basin in the equatorial hydroregion (where there is a large positive bias in the baseline streamflow). Horizontal axis shows the bias in the baseline streamflow and the vertical axis shows the normalized change (after calibration) for each parameter. Zero on y-axis indicates no change.

To understand how model parameters adjust to the bias in the baseline streamflow, we look at two hydro-regions with contrasting KGE skill scores. Equatorial and mid-latitude basins have predominantly large positive and negative streamflow biases respectively. Fig. 8 presents the relative change in model parameter values after calibration (normalized by the range) for equatorial basins. The normalized change for each parameter is defined as (P_c_-P_d_)/(P_max_-P_min_), where P_c_ is calibrated; P_d_ is default; P_max_ is maximum; and P_min_ is minimum parameter values (see Table 1). The most important parameter in improving the KGE in the equatorial basins (with predominantly positive bias) is ‘GwLoss’, which is a fraction of lower zone outflow transferred to deep groundwater storage and does not rejoin the river channel. This indicates that the calibration algorithm increases the groundwater loss parameter to correct for positive bias in the simulated streamflow. The KGE skill score is not as sensitive to changes in other parameters in the region.

**Fig. 9:**
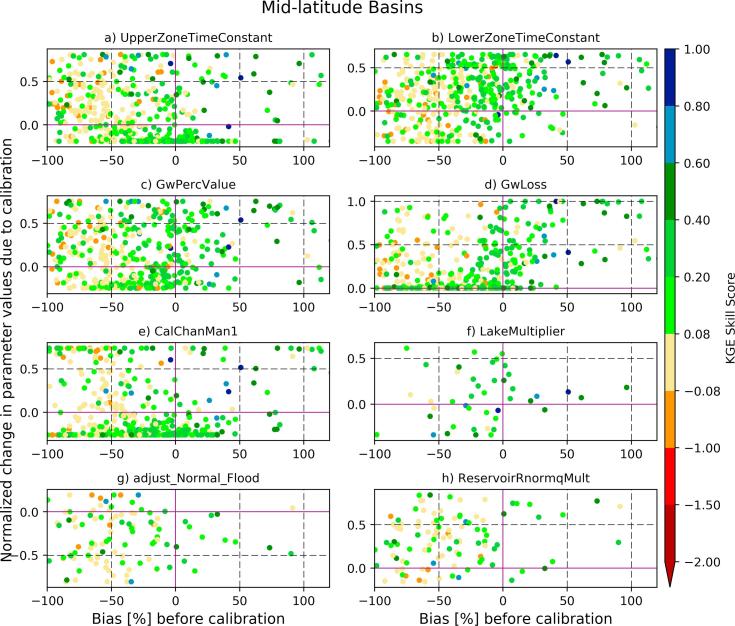
The same as Fig. 8 but for mid-latitude basins (with a significant negative bias in the baseline streamflow).

However, it is more challenging to improve the forecast skill when there is a water deficit in the simulated streamflow due to, for example, an underestimation in the runoff depth. The LISFLOOD model, under the current GloFAS setup, controls the timing of streamflow and the groundwater percolation, but it does not have any parameter to adjust for the deficit in the runoff depth. This is reflected in the mid-latitudes (Fig. 9) in which a large number of basins have negative streamflow bias. The KGE skill gain was obtained for the limited number of basins with negative streamflow bias by tuning the channel Manning's coefficient (using ‘*calChanMan1*′) perhaps through improving the flow timing (i.e., correlation). The deep groundwater loss parameter (‘GwLoss’) remains important for basins with positive bias in the region.

**Fig. 10:**
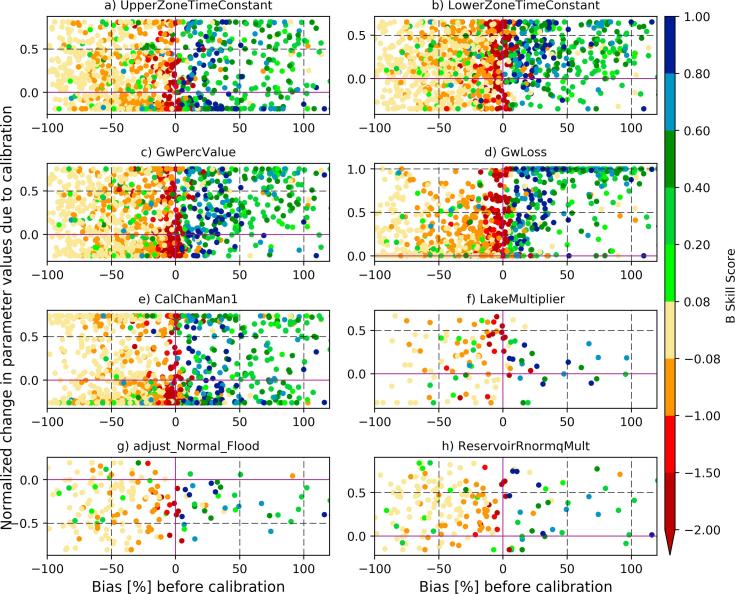
Streamflow bias skill score after calibration for all 1287 stations.

As a further step, we look at the bias skill score, i.e., the relative change in the streamflow bias compared to the baseline simulation. Even though bias was not explicitly used as an objective function in the calibration algorithm, it is still generally expected to improve since it is one component of the objective function (KGE, Gupta et al., 2009). Fig. 10 shows the impact of calibrating model parameters on reducing the streamflow bias. There is a clear separation in the bias skill score based on the baseline streamflow: the bias was reduced for basins with overestimation in baseline simulation and it was increased for basins with streamflow underestimation. Similar to the case for KGE skill score, the parameter responsible for the water transfer to deep groundwater is the main responsible for the bias skill improvement. The overall results indicate that the negative bias cannot be corrected with the 8 parameters currently used in LISFLOOD model calibration, and that methods to reduce the bias in the runoff volume (e.g., calibrating the land surface model) should be explored.

## 6 Discussions

The calibration of the LISFLOOD parameters resulted in a substantial improvement of simulated streamflow KGE score (both during the calibration and validation periods) for the majority of stations around the world. The KGE skill score varies across different regions mainly based on the bias in the baseline streamflow. On the one hand river basins with positive bias in the baseline simulation showed the largest improvements in the skill score after calibration and on the other hand those with negative bias showed the lowest skill score.

There are possible reasons for the low skill score for some of the river basins worldwide (4% of the 1287 stations had negative KGE skill score). Firstly, it should be noted that the parameters for the land surface model (H-TESSEL), responsible for the partitioning of precipitation into runoff and evapotranspiration, were not calibrated in this work. This means that only the parameters related to the flow timing, variability and groundwater loss were altered during the calibration process, without changing other hydrological model parameters (such as those related to evaporation, infiltration, etc.). This limits the effectiveness of the calibration algorithm for basins where, for example, forcing bias is the main source of streamflow uncertainty. Secondly, uncertainties in the observed streamflow due to errors in station sites, data collection, human intervention, and other sources should always be taken into account (e.g., Wilby et al., 2017). Careful manual inspection and corrections (e.g., locating on the river channel, matching upstream area between provided and GloFAS estimated) have been performed for the stations used in this work; however the quality control of multi-source, diverse instrumentation global-scale hydrological dataset remains a challenging task. Thirdly, uncertainty in the forcing data may result in fitting model parameters to an erroneous input data. This could introduce additional errors to model parameters and could reduce the accuracy of streamflow simulation during the validation period. Finally, the calibration skill loss may partly be attributed to a premature stoppage of the evolutionary algorithm. Some basins may need longer than the maximum 372 simulations (15 generations) set in this work to converge to a global maximum.

The following further steps can be taken to improve the skill score. Firstly, the bias in the surface and subsurface runoff should be reduced. This can be done through improving the skill in the meteorological (mainly precipitation) forecasts and by calibrating the land surface model (H-TESSEL, e.g., Orth et al., 2016). Calibration of the land surface model parameters, not just the LISFLOOD model parameters, may offset the large bias in precipitation forcing. Secondly, regionalization of model parameters can be applied to transfer model parameters from best performing basins to basins with similar characteristics, but with poor scores or with limited streamflow data (e.g., Beck et al., 2016, Samaniego et al., 2010). Finally, multiple objective functions (to account for the high flows) can be used and the EA calibration algorithm can be run for longer generations to prevent a potential premature stoppage. Longer runs with different stopping criteria (see Maier et al., 2014) than those implemented in this work may result in better solutions. This is, however, dictated by the time demands of such a computationally demanding global scale calibration.

The distribution of streamflow observations available for use in this calibration study is unevenly distributed around the world, leaving GloFAS uncalibrated for several flood-prone rivers in developing regions. For example, major river basins in Asia with history of frequent flooding (e.g., Indus, Ganges, Brahmaputra, and Mekong) are not included in this study. To fill the gaps in streamflow observations, future calibration work could utilize satellite-based river level or width estimations (e.g., Revilla-Romero et al., 2015a, Andreadis et al., 2007, Brakenridge et al., 2007).

## 7 Conclusions

This work is a first attempt to calibrate the model underpinning GloFAS operational flood forecasts using daily streamflow data from 1287 stations around the world. An evolutionary algorithm with KGE as an objective function was applied. ECMWF reforecast was used as model forcing and a combination of improvement based criteria and exhaustion-based criteria were employed for stopping the calibration algorithm. Results show that calibrating the routing and groundwater model improved streamflow simulations (KGE skill score) compared with the baseline (with default parameters) for the large majority of stations. However, the skill gained varies depending on the bias in the baseline simulation: the largest improvements occurred in areas with positive bias in the baseline, while the lowest skill score was obtained in basins with a negative bias. The disparate sensitivity of skill scores to model parameters suggests that calibrating the routing and groundwater model parameters is an important step but it is not sufficient for reducing the streamflow forecast uncertainty. Future efforts in improving the accuracy of GloFAS flood forecasts could mainly focus on improving the global runoff estimation through reducing the errors in the meteorological forecasts (such as bias reduction) and improving the land surface model performance. In addition, explicitly including an objective function that is exclusively based on peak flows could potentially improve the flood forecasting.

## References

[b0005] Alfieri L., Burek P., Dutra E., Krzeminski B., Muraro D., Thielen J., Pappenberger F. (2013). GloFAS—Global ensemble streamflow forecasting and flood early warning. Hydrol. Earth Syst. Sci..

[b0010] Alfieri L., Pappenberger F., Wetterhall F. (2014). The extreme runoff index for flood early warning in Europe. Nat. Hazards Earth Syst. Sci..

[b0015] Andreadis K.M., Clark E.A., Lettenmaier D.P., Alsdorf D.E. (2007). Prospects for river discharge and depth estimation through assimilation of swath-altimetry into a raster-based hydrodynamics model. Geophys. Res. Lett..

[b0020] Balsamo G., Beljaars A., Scipal K., Viterbo P., van den Hurk B., Hirschi M., Betts A.K. (2009). A revised hydrology for the ECMWF model: Verification from field site to terrestrial water storage and impact in the integrated forecast system. J. Hydrometeor., 10, 623–643, doi:10.1175/2008JHM1068.1.Balsamo, G.A., and Coauthors, 2015: ERA-Interim/Land: A global land water resources dataset. Hydrol. Earth Syst. Sci..

[b0025] Beck H.E., van Dijk A.I.J.M., deRoo A., Miralles D.G., McVicar T.R., Schellekens J., Bruijnzeel L.A. (2016). Global-scale regionalization of hydrologic model parameters. WaterResour. Res..

[b0030] Beck H.E., Vergopolan N., Pan M., Levizzani V., van Dijk A.I.J.M., Weedon G.P., Brocca L., Pappenberger F., Huffman G.J., Wood E.F. (2017). Global-scale evaluation of 22 precipitation datasets using gauge observations and hydrological modeling. Hydrol. Earth Syst. Sci..

[b0035] Biemans H., Hutjes R.W.A., Kabat P., Strengers B., Gerten D., Rost S. (2009). Effects of precipitation uncertainty on discharge calculations for main river basins. J. Hydrometeorol..

[b0040] Bierkens, Marc F. P., 2015. Global Hydrology 2015: State, Trends, and Directions. Water Resour. Res. 51(7) 4923–47. doi:10.1002/2015WR017173.

[b0045] Bollrich G. (1992). Technische Hydromechanik: Grundlagen/Gerhard Bollrich; Günter Preissler.

[b0050] Brakenridge G.R., Nghiem S.V., Anderson E., Mic R. (2007). Orbital microwave measurement of river discharge and ice status. Water Resour. Res..

[b0055] Burek, P., Van Der Knijff, J., De Roo, A., 2013. LISFLOOD, distributed water balance and flood simulation model: Revised user manual. JRC Tech. Rep., p. 138.

[b0060] Chow V.T., Maidment D.R., Mays L.M. (1988). Applied hydrology.

[b0065] Dadson S.J., Bell V.A., Jones R.G. (2011). Evaluation of a grid-based river flow model configured for use in a regional climate model. J. Hydrol..

[b0070] De Rainville D.M., Fortin F.A., Gardner M.A., Parizeau M., Gagné C. (2014). DEAP: enabling nimbler evolutions. ACM SIGEVOlution.

[b0075] Deb K., Pratab A., Agarwal S., Meyarivan T. (2002). A fast elitist non-dominated sorting genetic algorithm for multi-objective optimization: NSGA-II. IEEE Trans. Evol. Comput..

[b0080] Decharme B., Alkama R., Douville H., Becker M., Cazenave A. (2010). Global Evaluation of the ISBA-TRIP Continental Hydrological System. Part II: Uncertainties in River Routing Simulation Related to Flow Velocity and Groundwater Storage. J. Hydromet*.*.

[b0085] Döll P., Douville H., Güntner A., Müller Schmied H., Wada Y., Cazenave A., Champollion N., Benveniste J., Chen J. (2016). Modelling Freshwater Resources at the Global Scale: Challenges and Prospects. Remote Sensing and Water Resources.

[b0090] ECMWF (2017). Changes in ECMWF model. https://www.ecmwf.int/en/forecasts/documentation-and-support/changes-ecmwf-model.

[b0095] Elsner M.M., Gangopadhyay S., Pruitt T., Brekke L.D., Mizukami N., Clark M.P. (2014). How does the choice of distributed meteorological data affect hydrologic model calibration and streamflow simulations?. J. Hydrometeor..

[b0100] Emerton R.E., Stephens E.M., Pappenberger F., Pagano T.C., Weerts A.H., Wood A.W., Salamon P., Brown J.D., Hjerdt N., Donnelly C., Baugh C.A., Cloke H.L. (2016). Continental and Global Scale Flood Forecasting Systems. WIREs Water, in press.

[b0105] Fekete B.M., Vörösmarty C.J., Roads J.O., Willmott C.J. (2004). Uncertainties in precipitation and their impacts on runoff estimates. J. Climate.

[b0110] Feyen Luc, Vrugt Jasper A., Nualláin Breanndán Ó, van der Knijff Johan, De Roo Ad. (2007). Parameter Optimisation and Uncertainty Assessment for Large-Scale Streamflow Simulation with the LISFLOOD Model. J. Hydrol..

[b0115] Fortin F., De Rainville F., Gardner M., Parizeau M., Gagne C. (2012). DEAP: Evolutionary algorithms made easy. J. Mach. Learn. Res..

[b0120] Goteti G., Famiglietti J.S., Asante K. (2008). A Catchment-Based Hydrologic and Routing Modeling System withexplicit river channels. J. Geophys. Res..

[b0125] Gupta H.V., Kling H., Yilmaz K.K., Martinez G.F. (2009). Decomposition of the mean squared error and NSE performance criteria: implications for improving hydrological modelling. J. Hydrol..

[b0130] Hadka D., Reed P. (2013). Borg: an auto-adaptive many-objective evolutionary computing framework. Evol. Comput..

[b0135] Hirpa F.A., Salamon P., Alfieri L., Thielen-del Pozo J., Zsoter E., Pappenberger F. (2016). The effect of reference climatology on global flood forecasting. J. Hydromet..

[b0140] Hirpa, F.A., et al., 2018. Global Flood Forecasting for Averting Disasters Worldwide, chapter 12 in “Global Flood Hazard: Applications in modeling, mapping and forecasting”. In: Guy Schumann, Paul Bates, Giuseppe Aronica, Heiko Apel (eds.) Wiley, https://doi.org/10.1002/9781119217886.ch12.

[b0145] ICOLD, 2009. International Commission on Large Dams, World Register of Dams. Version update 2009. ICOLD, Paris, France. www.icold-Cigb.net.

[b0150] Kang S., Ahn J.-B. (2015). Global energy and water balances in the latest reanalyses. Asia-Pacific J. Atmos. Sci..

[b0155] Khu S.T., Madsen H., di Pierro F. (2008). Incorporating multiple observations for distributed hydrologic model calibration: an approach using a multi-objective evolutionary algorithm and clustering. Adv. Water Resour..

[b0160] Kollat J.B., Reed P. (2005). Comparing State-of-the-Art Evolutionary Multi-Objective Algorithms for Long-Term Groundwater Monitoring Design. Adv. Water Resour..

[b0165] Lehner B., Döll P. (2004). Development and validation of a globaldatabase of lakes, reservoirs and wetlands. J. Hydrol..

[b0170] Lehner B., Liermann C.R., Revenga C., Vörösmarty C., Fekete B., Crouzet P., Döll P. (2011). High-resolution mapping of the world’s reservoirs and dams for sustainable river-flow management. Front. Ecol. Environ..

[b0175] Li H., Luo L., Wood E.F., Schaake J. (2009). The role of initial conditions and forcing uncertainties in seasonalhydrologic forecasting. J. Geophys. Res..

[b0180] Li H., Wigmosta M.S., Wu H., Huang M., Ke Y., Coleman A.M., Leung L.R. (2013). A physically based runoff routing model for land sur-face and earth system models. J. Hydrometeorol..

[b0185] Maier H.R., Co-authors (2014). Evolutionary algorithms and other metaheuristics in water resources: Current status, research challenges and future directions. Environ. Modell. Software.

[b0190] Martí L., García J., Berlanga A., Molina J.M. (2009). An approach to stopping criteria for multi-objective optimization evolutionary algorithms: the MGBM criterion. IEEE Congress On Evolutionary Computation (CEC’09).

[b0195] Materia S., Dirmeyer P.A., Guo Z., Alessandri A., Navarra A. (2010). The sensitivity of simulated river discharge to land surface representation and meteorological forcings. J. Hydrometeor..

[b0200] Muleta M.K., Nicklow J.W. (2005). Sensitivity and uncertainty analysis coupled with automatic calibration for a distributed watershed model. J. Hydrol..

[b0205] Müller Schmied H., Eisner S., Franz D., Wattenbach M., Portmann F.T., Flörke M., Döll P. (2014). Sensitivity of simulated global-scale freshwater fluxes and storages to input data, hydrological model structure, human water use and calibration. Hydrol. Earth Syst. Sci..

[b0210] Meybeck M., Kummu M., Dürr H.H. (2013). Global hydrobelts and hydroregions: improved reporting scale for water-related issues?. Hydrol. Earth Syst. Sci..

[b0215] Nasonova O.N., Gusev Y.M., Kovalev Y.E. (2011). Impact of uncertainties in meteorological forcing data and land surface parameters on global estimates of terrestrial water balance components. Hydrol. Process..

[b0220] Nicklow J.W., Reed P.M., Savic D., Dessalegne T., Harrell L., Chan-Hilton A. (2010). 2010: State of the art for genetic algorithms and beyond in water resources planning and management. J. Water Resour. Plan Manage.

[b0225] Orth R., Dutra E., Pappenberger F. (2016). Improving Weather Predictability by Including Land Surface Model Parameter Uncertainty. Monthly Weather Rev..

[b0230] Pappenberger F., Thielen J., Del Medico M. (2011). The impact of weather forecast improvements on large scale hydrology: analysing a decade of forecasts of the European Flood Alert System. Hydrol. Process..

[b0235] Revilla-Romero B., Beck H.E., Burek P., Salamon P., de Roo A., Thielen J. (2015). Filling the gaps: Calibrating a rainfall-runoff model using satellite-derived surface water extent. Remote Sens. Environ..

[b0240] Revilla-Romero B., Hirpa F.A., Pozo J.T.-D., Salamon P., Brakenridge R., Pappenberger F., De Groeve T. (2015). On the Use of Global Flood Forecasts and Satellite-Derived Inundation Maps for Flood Monitoring in Data-Sparse Regions. Remote Sens..

[b0245] Samaniego L., Kumar R., Attinger S. (2010). Multiscale parameter regionalization of a grid-based hydrologic model at the mesoscale. Water Resour. Res..

[b0250] Sampson C.C., Fewtrell T.J., O’Loughlin F., Pappenberger F., Bates P.B., Freer J.E., Cloke H.L. (2014). The impact of uncertain precipitation data on insurance loss estimates using a flood catastrophe model. Hydrol. Earth Syst. Sci..

[b0255] Sperna Weiland F.C., Vrugt J.A., van Beek R.P.H., Weerts A.H., Bierkens M.F.P. (2015). Significant uncertainty in global scale hydrological modeling from precipitation data errors. J. Hydrol..

[b0260] Tang Y., Reed P., Wagener T. (2006). How effective and efficient are multiobjective evolutionary algorithms at hydrologic model calibration?. Hydrol. Earth Syst. Sci..

[b0265] Trenberth K.E., Fasullo J.T., Mackaro J. (2011). Atmospheric moisture transports from ocean to land and global energy flows in reanalyses. J. Clim..

[b0270] Werth S., Güntner A. (2010). Calibration analysis for water storage variability of the global hydrological model WGHM. Hydrol. Earth Syst. Sci..

[b0275] Wi S., Yang Y.C.E., Steinschneider S., Khalil A., Brown C.M. (2015). Calibration approaches for distributed hydrologic models in poorly gaged basins: Implication for streamflow projections under climate change. Hydrol. Earth Syst. Sci..

[b0280] Wilby R., Clifford N., De Luca P., Harrigan S., Hillier J., Hodgkins R., Matthew T., Murphy C., Noone S., Parry S., Prudhomme C., Rice S., Slater L., Smith K., Wood P. (2017). The “dirty dozen” of freshwater science: detecting then reconciling hydrological data biases and errors. Wiley Interdisciplinary Reviews (WIREs) Water.

[b0285] Wu H., Kimball J.S., Li H., Huang M., Leung L.R., Adler R.F. (2012). A new global river network database for macroscale hydrologic modeling. Water Resour. Res..

[b9000] Wu H., Adler R.F., Tian Y., Huffman G.J., Li H., Wang J. (2014). Real-time global flood estimation using satellite-based precipitation and a coupled land surface and routing model. Water Resour. Res..

[b0290] Xue X., Zhang K., Hong Y., Gourley J.J., Kellogg W., McPherson R.A., Wan Z., Austin B.N. (2016). New Multisite Cascading Calibration Approach for Hydrological Models: Case Study in the Red River Basin Using the VIC Model. J. Hydrol. Eng..

[b0295] Yamazaki D., O'Loughlin F., Trigg M.A., Miller Z.F., Pavelsky T.M., Bates P.D. (2014). Development of the Global Width Database for Large Rivers. Water Resour. Res..

[b0300] Zajac Z., Revilla-Romero B., Salamon P., Burek P., Hirpa F., Beck H. (2017). The impact of lake and reservoir parameterization on global streamflow simulation. J. Hydrol..

[b0305] Zsoter E., Pappenberger F., Smith P., Emerton R.E., Dutra E., Wetterhall F., Richardson D., Bogner K., Balsamo G. (2016). Building a multimodel flood prediction system with the TIGGE archive. J. Hydrometeorol..

